# Characterization of the Fouling Layer on the Membrane Surface in a Membrane Bioreactor: Evolution of the Foulants’ Composition and Aggregation Ability

**DOI:** 10.3390/membranes9070085

**Published:** 2019-07-16

**Authors:** Linlin Yan, Ruixue Li, Yu Song, Yanping Jia, Zheng Li, Lianfa Song, Haifeng Zhang

**Affiliations:** 1 School of Chemistry Engineering, Northeast Electric Power University, Jilin 132012, Jilin, China; 2 Department of Civil and Environmental Engineering, Texas Tech University, 10th and Akron, Lubbock, TX 79409-1023, USA

**Keywords:** membrane bioreactor, transmembrane pressure, membrane foulants, aggregation, extracellular polymeric substances

## Abstract

In this study, the characteristics of membrane foulants were analyzed with regard to morphology, composition, and aggregation ability during the three stages of transmembrane pressure (TMP) development (fast–slow–fast rise in TMP) in a steady operational membrane bioreactor (MBR). The results obtained show that the fouling layer at the slow TMP-increase stage possessed a higher average roughness (71.27 nm) and increased fractal dimension (2.33), which resulted in a low membrane fouling rate (0.87 kPa/d). A higher extracellular DNA (eDNA) proportion (26.12%) in the extracellular polymeric substances (EPS) resulted in both higher zeta potential (-23.3 mV) and higher hydrophobicity (82.3%) for initial foulants, which induced and increased the protein proportion in the subsequent fouling layer (74.11%). Furthermore, the main composition of the EPS shifted from protein toward polysaccharide dominance in the final fouling layer. The aggregation test confirmed that eDNA was essential for foulant aggregation in the initial fouling layer, whereas ion interaction significantly affected foulant aggregation in the final fouling layer.

## 1. Introduction

Membrane bioreactors (MBRs), as an attractive solution for wastewater treatment and reuse, provide many advantages over conventional activated sludge systems, for example, a smaller footprint and better effluent quality, etc. [1,2]. Nevertheless, the biofouling has become a major bottleneck for the wide application of MBRs [3]. According to the variations of the transmembrane pressure (TMP), the biofouling in MBRs can be divided into three stages. At the initial stage, the extracellular polymeric substances (EPS) firstly adhere onto the membrane surface, resulting in a rapid increase in TMP. The second stage is characterized by a slow increase in TMP due to the accumulated foulants. Then, the TMP begins to increase more rapidly at a high fouling rate [4,5]. Several authors reported that the cake layer was considered as the dominant foulant and the cake resistance was the major membrane resistance, accounting for about 85% of total resistance [6,7]. In general, EPS have been recognized as the main fouling components of the fouling layer in MBRs, resulting in an increase in TMP [8,9]. Therefore, the dynamic variations of EPS composition have a significant correlation with the TMP development.

The EPS is composed of polysaccharides (PS), proteins (PN), extracellular DNA (eDNA), and metal ions, etc. [7]. The current consensus suggests that the PN and PS are the major factions in EPS that contribute to biofouling, however, other components may also play central roles. For example, eDNA has been reported to function as a scaffold, which provides structural integrity to the EPS matrix. It can introduce favorable acid–base interactions that are responsible for bacterial aggregation and adhesion to the surface in conventional biofilm studies [10,11]. Until now, the detailed roles of the EPS components in biofouling were unexplored, especially with regard to eDNA [12]. Information about the changes of EPS components in response to the TMP development could help to identify the specific EPS component that is responsible for biofouling. 

The fouling layer formation in MBRs is a thermodynamic process [13]. The resulting interaction energy between foulants and membrane can be divided into two types: (1) the attachment strength between initial foulants and the virgin membrane; and (2) the self-aggregation strength between foulants [14,15]. Matar et al. reported that the initial fouling layer developed quickly on the different types of membrane surfaces and then the fouling selection of the membrane-surface chemistry reduced since the membrane lost its intrinsic affinity [8]. Therefore, further understanding of the self-aggregation ability between foulants is indispensable to mitigate membrane fouling.

Therefore, the aim of this study was to investigate the evolution of the EPS composition as a TMP development and assess their corresponding aggregation ability. Membrane foulants were collected from the membrane surface following different operational time periods in a lab-scale MBR. The EPS composition was characterized in terms of PN, PS, and eDNA content. Deoxyribonuclease (DNase) and ethylene diamine tetraacetic acid (EDTA) were used to specially target eDNA and metal ions in each sample separately to identify the role of the EPS component in the aggregation of membrane foulants. 

## 2. Material and Methods

### 2.1. Experimental MBR Setup 

**Figure 1:**
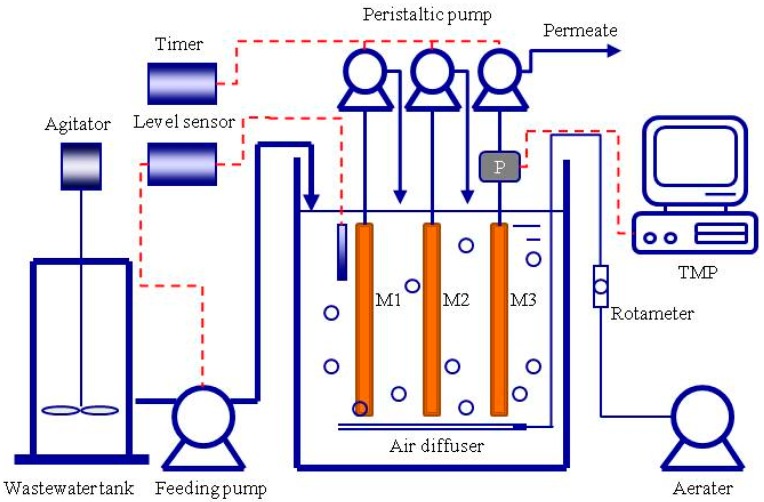
Schematic diagram of the membrane bioreactor (MBR) experimental setup. M1, M2, and M3 are identical membrane modules; TMP stands for transmembrane pressure; P stands for pressure meter.

A lab-scale submerged MBR was operated with a working volume of 7.5 L (Figure 1). Three identical membrane modules named M1, M2, and M3 were vertically mounted within the MBR tank. They were polyvinylidene fluoride (PVDF) flat sheet microfiltration membranes with a filtration area of 0.1 m^2^ per module (Nanjing Tengxiang Co. Ltd., Nanjing, China). The nominal membrane pore diameter was 0.1 μm. Air was supplied to the reactor at 400 L/h.

### 2.2. Operation Conditions

The MBR was seeded with 5 g/L of the acclimatized activated sludge. The seed sludge was taken from another lab-scale MBR that had been operated for more than two years. The hydraulic retention time (HRT) and sludge retention time (SRT) were maintained at 8.6 h and 30 days, respectively. The membrane modules were run in parallel using the same permeate flux of 10 L/m^2^·h. The permeate through each membrane module was continuously withdrawn using a peristaltic pump (Model BT-100, Baoding Longer Precision Pump Co., Ltd., Baoding, China), utilizing a 13 min on and 2 min off mode. In order to maintain the predetermined HRT, the permeates of M1 and M2 were recycled back to the MBR tank. Synthetic wastewater was used as the nutrient mixture for the MBR biomass. The average concentrations of chemical oxygen demand (COD), total nitrogen (TN), and total phosphorous (TP) were about 310, 30, and 7 mg/L, respectively. The recipe can be found in our previous study [2]. Prior to carrying out the experiment, the MBR was operated for 60 days to achieve the stable stage, both COD and NH_4_^+^-N were steadily removed in the MBR regardless of the influent conditions, maintaining average removal rates above 92.5% and 90.7%, respectively. The mixed liquor suspended solid (MLSS) and mixed liquor volatile suspended solid (MLVSS) concentrations ranged from 8–9 and 6–7.5 g/L, respectively. 

### 2.3. Analytic Methods

#### 2.3.1. Sample Collection and Biofilm Treatment

The membrane module was carefully removed from the MBR tank at different operational time points (days 1, 5, and 11) and fresh foulants were scraped from the membrane surface with a blade. The collected sample was suspended in 50 mL ultrapure water. The foulant solution was then dispersed for 10 min using a vortex mixer (XW-80A, Shanghai Tangchi Electronics Co., Ltd., Shanghai, China) at full speed. 

DNase (DN25-10MG, Sigma Aldrich Trading Co., Ltd., Shanghai, China) and EDTA (EDS-100G, Sigma Aldrich Trading Co., Ltd., Shanghai, China) were separately used to specially target eDNA and metal ions in each sample. The foulant solution of the first group was treated with eDNA hydrolytic enzyme and 170 U of DNase was added to 10 mL of the biofilm solution for 5 h at 55 °C under moderately agitated conditions [16]. The sample of the second group was treated with a cation chelating agent and 0.1 mg of EDTA was added to 10 mL of the foulant solution and incubated at 55 °C for 5 h under agitated conditions [17]. As a control, a similar sample incubation was conducted for the foulant sample without any treatment.

#### 2.3.2. EPS Extract and Determination

In this study, total EPS refers to soluble EPS and the non-soluble fraction of the EPS, which were extracted according to Wei et al. [18]. PS was measured using the phenol-sulfuric acid method with glucose as the standard (Sinopharm) [19]. PN was determined via the modified Lowry method using bovine serum albumin (BSA) as the standard (Sigma) [20]. eDNA was determined via the diphenylamine colorimetric method using fish sperm DNA as the standard (Sigma) [21].

#### 2.3.3. Aggregation Test

Aggregation assays were conducted using the developed method described by Eboigbodin and Biggs [22], with minor modifications. Briefly, the pellets were resuspended in EPS solutions with and without treatments. The *OD*_600,__0_ of the cell suspensions was adjusted to ~0.6. Then, 2 mL of the suspension was transferred into the cuvette. After 5 h, the *OD*_600,__5_ at the upper part of the cuvette was determined. All assays were performed in duplicate. The aggregation efficiency (*A*_e_) of biofilm cells was calculated using Equation (1).

(1) $$Ae=\frac{OD_{600,0}-OD_{600,5}}{OD_{600,0}}\times 100$$

#### 2.3.4. Other Analytic Methods

The relative hydrophobicity of foulant samples was measured as described by Pembrey et al. [23]. The zeta potentials of samples were recorded using a Zetasizer Nano ZS Instrument (Malvern, Malvine, UK). Atomic force microscopy (AFM) images (not shown) were taken with the non-contact mode (XE7, Park, Suwon, Korea). The average roughness and the fractal dimension were obtained from XEI software provided by the AFM manufacturer. Standard analytic methods were used to measure the COD, NH_4_^+^-N, MLSS, and MLVSS [24].

## 3. Results and Discussion

### 3.1. Change of Surface Morphology during the Three Phases 

**Figure 2:**
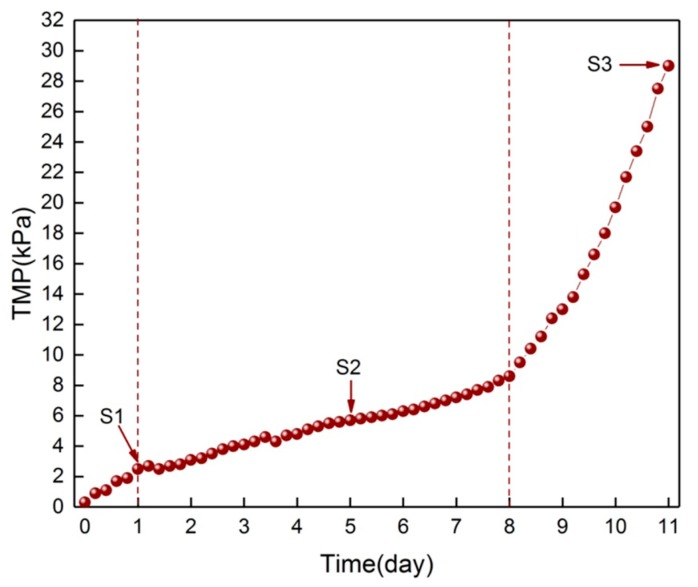
Change of the TMP during M3 operation. S1, S2, and S3 are samples scraped from M1, M2, and M3, respectively.

The variations of the TMP over the long-term operation of M3 are shown in Figure 2, and the corresponding three samples scraped from M1, M2, and M3 were designated S1, S2, and S3. The results clearly indicate that the TMP showed a three-phase evolution. At the start of operation, the TMP showed an initial increasing rate of 2.2 kPa/d. Then, the TMP increasing rate slowed down to 0.87 kPa/d from day 1 to day 8. After a slow TMP increasing stage, a sudden TMP increase at a rate of 8.6 kPa/d lasted for about 3 days. Similar TMP increases with the three-phase increasing modes were reported previously [4,5]. The membrane pores of the virgin membrane can clearly be seen in the SEM image. While the surface of M1 was completely covered with a thin layer, the collected foulants were a somewhat transparent slime. With respect to the M2 surface, a lot of foulants accumulated on the membrane surface. The fouling layer was more compact near the membrane surface and looser on the outer layer. Further adhesion of foulants was observed on the M3 surface, which was more heterogeneous compared to M2 and formed a stable three-dimensional structure.

The parameters associated with surface morphologies of the virgin membrane, as well as M1, M2, and M3 are listed in the Table 1. A slight increase in average roughness was observed for the M1 surface (38.54 nm) compared to the virgin membrane (37.35 nm), indicating that the initial foulants seemed to be flexible and tended to fit the membrane surface topography. At the slow TMP-increasing stage, the average roughness increased significantly for M2 compared to M1. The roughness increased by 41.41% and the fouling surface became ragged and exhibited clear valleys and peaks. Lin et al. reported that the rough membrane surface showed weaker interaction strength with the foulants [25], which would be a reason for the low fouling rate (0.87 kPa/d) at this stage. Compared to M2, the final fouling layer surface showed peaks and valleys combined with each other which led to a flatter M3 surface and decreasing roughness (from 71.27 to 53.28 nm).

The fractal dimension (*D_f_*) is considered as a helpful parameter for the complexity characterization of membrane surfaces [26]. The lower *D_f_* of the surface indicates increased stickiness and easy adhesion of foulants in the MBR [27]. *D_f_* values could be ordered in the following way: virgin membrane (2.35) > M2 (2.33) > M3 (2.31) > M1 (2.25) (Table 1), which suggests that, at any stage, the fouling layer exhibits a higher fouling potential compared to the virgin membrane. Moreover, a lower *D_f_* was found in the M2 surface, implying that the accumulated foulants at the slow TMP-rise stage had a lower fouling potential compared to the foulants on the M1 or M3 surface.

### 3.2. Temporal Dynamics of EPS Composition during Biofouling Layer Development 

**Figure 3:**
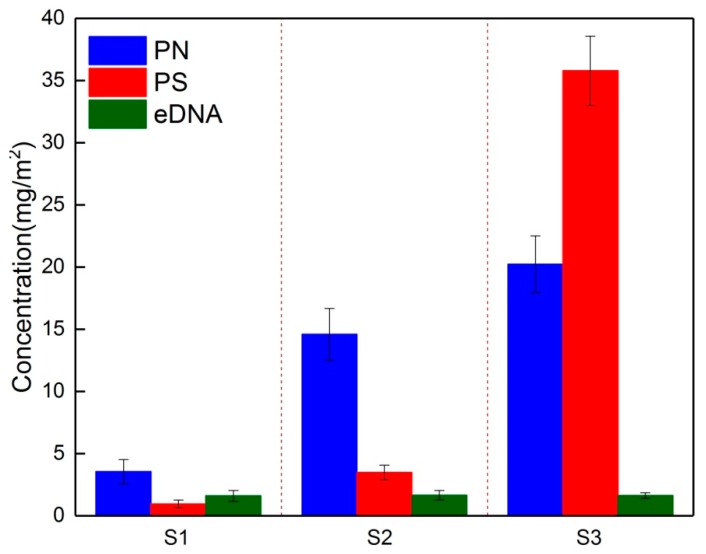
Variations of extracellular polymeric substances (EPS) composition in the biofouling layer on the membrane surfaces. PN = proteins; PS = polysaccharides; eDNA = extracellular DNA.

Quantitative analysis of the PN, PS, and eDNA within EPS during the fouling developmental stages and their proportions in total EPS are shown in Figure 3. Both PN and PS contents increased with the operating time, whereas the eDNA amount remained at a stable level. Specifically, the eDNA content was 1.59 mg/m^2^ in the initial fouling layer (S1), which accounted for the highest proportion (26.12%) in total EPS compared to S2 or S3 (8.32% for S2 and 2.43% for S3), implying that eDNA had high adhesive fouling to the virgin membrane surface. 

Correspondingly, the high proportions of eDNA in the EPS led to a higher absolute value of zeta potential (−23.3 mV) and higher hydrophobicity (82.3%) for S1 (see Table 2). In fact, eDNA contains a large amount of deoxyribose moieties and phosphate groups, the former leads to an increase in surface hydrophobicity, while the latter enhances its electronegativity [28].

A significant increase in PN was observed for S2, which constituted about 74.11% of the total EPS. Higher PN content on the membrane surface during the steady membrane fouling stage was also reported in a previous report [8]. Matar et al. investigated the temporal changes in EPS on hydrophobic and hydrophilic membrane surfaces in a lab-scale MBR; they found that the same type of organic foulants developed over time on the different membrane surface and the PN dominated the EPS components [8]. Regardless of microbe metabolism within the biofouling layer, the increment of PN at this stage was likely mainly caused by adsorption from the buck liquor by the initial fouling layer. Since S1 possessed a more negative charge and higher hydrophobicity, it had a high potential to bind amino groups in the PN, resulting in a high PN content in S2. The increment of PN in S2 caused a lower absolute value of zeta potential (−18.7 mV) and a higher hydrophobicity (88.1%). An obvious increase in PS content was observed in the final fouling layer (S3), which contributed 62.4% to the total EPS. The increase in PS content in the final fouling layer led to poor hydrophobicity (56.3%). Moreover, the increment of the Ca^2+^ content in S3 decreased the absolute value of the zeta potential (−14.8 mV). From the three-stage fouling process, the different EPS components dominated the biofouling layer over time, for example, a higher PN and eDNA content was found in the initial fouling layer (S1), and the main composition of the EPS shifted from PN (S2) towards PS dominance in the final biofouling layer (S3).

### 3.3. Key Component in EPS for the Aggregation Ability of Foulants

**Figure 4:**
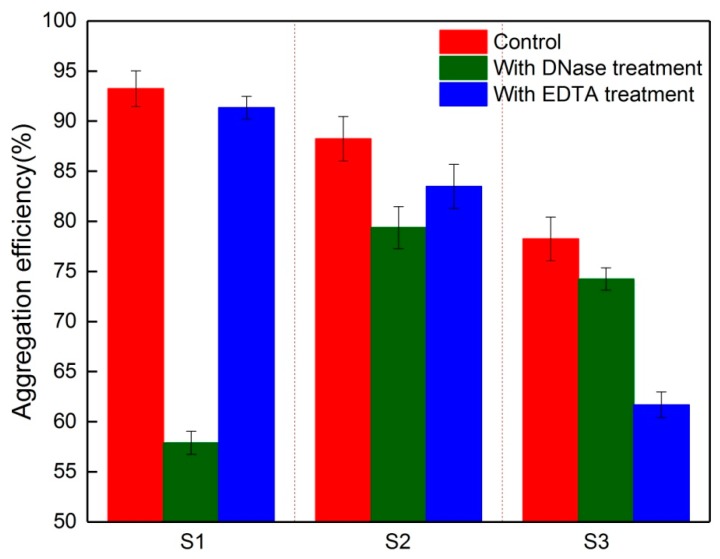
Aggregation efficiency of foulants without treatment after deoxyribonuclease (DNase) or ethylene diamine tetraacetic acid (EDTA) treatment.

Figure 4 shows the aggregation ability of S1, S2, and S3 with and without treatment by DNase or EDTA. The EPS in S1 without treatment had the greatest aggregation ability (93.2% on average). Removal of eDNA via DNase treatment dramatically reduced the aggregation ability of S1 (57.9% on average), which demonstrated that eDNA was essential for aggregation in the initial biofilm [11]. The role of eDNA in aggregation decreased with biofilm development, for example, the aggregation efficiency decreased from 88.2% for S2 without treatment to 79.4% with eDNA treatment. Regardless of eDNA, the presence of abundant PN in S2 might promote the aggregation ability of foulants. Furthermore, it was reported that hydrophobic PN were responsible for the critical role of hydrogen bonds in microbial aggregation [12]. These results indicate that the controlled membrane fouling by DNase for the initial fouling stage would be more effective. Das et al. also reported a similar result; they found that DNase addition resulted in the non-specific hydrolysis of phosphodiester bonds in DNA and the initial biofilm disintegration [11], whereas the DNase had no significant effect on the structural integrity of the mature biofilms [29].

The membrane foulants after EDTA treatment had little effect on the aggregation efficiency of S1 and S2. In contrast, a significantly decrease in aggregation ability was observed for S3 compared to the control, which indicated that the aggregation ability for S3 was inhibited as a result of the interrupting ion interactions by EDTA. Considering the high PS and Ca^2+^ content in S3 (see Figure 3 and Table 2), Ca^2+^ bridging appears to be more specific for PS than PN. Huang et al. established that Ca^2+^ had a positive effect on PS cohesion [30], which would improve the role of PS in EPS aggregation. The findings presented here indicate that the Ca^2+^ interaction significantly affected the aggregation of S3, and thus a cleaning agent with EDTA and acid would have a higher efficiency for membrane cleaning at this stage. Compared to EDTA, the acid cleaning would lead to secondary pollution via the discharge, increasing the potential environmental risk [31]. As a result, EDTA has a high potential for application in membrane cleaning.

## 4. Conclusions

Membrane foulants of the lab-scale submerged MBR were characterized during the three stages of TMP in this study. The results showed that a higher average roughness and fractal dimension of the fouling layer resulted in a low membrane fouling rate. The eDNA had a central role in the initial fouling layer formation, which induced the increment of PN in the subsequent fouling layers. The main composition of EPS shifted from PN toward PS dominance in the final fouling layer. The eDNA was essential for the initial foulants’ aggregation, whereas ion interaction played a significant role in foulants’ aggregation for the final fouling layer. To control membrane fouling, the cleaning agent with the DNase and EDTA was effective for the initial and final fouling layers, respectively. The information obtained from the lab-scale MBR in this work is essential for the establishment of a better understanding of the fouling layer development and is potentially applicable for membrane cleaning in MBR.

## Figures and Tables

**Table 1:** Roughness parameters and fractal dimensions during the different membrane fouling stages.

Item	Virgin Membrane	M1	M2	M3
Average roughness (nm)	37.35 ± 2.76	38.54 ± 1.25	71.27 ± 10.73	53.28 ± 3.65
Fractal dimension (*D_f_*)	2.35	2.25	2.33	2.31

**Table 2:** Zeta potential, hydrophobicity, and Ca^2+^ content for membrane foulants at different stages.

Item	S1	S2	S3
Zeta potential (mV)	−23.3	−18.7	−14.8
Hydrophobicity (%)	82.3	88.1	56.3
Ca^2+^ content (mg/g SS)	1.3	4.7	23.6
